# Determination of changes in the microbial and chemical composition of Țaga cheese during maturation

**DOI:** 10.1371/journal.pone.0242824

**Published:** 2020-12-03

**Authors:** Adriana Criste, Lucian Copolovici, Dana Copolovici, Melinda Kovacs, Robert H. Madden, Nicolae Corcionivoschi, Ozan Gundogdu, Mihaela Berchez, Adriana Cristina Urcan

**Affiliations:** 1 Department of Microbiology and Immunology, Faculty of Animal Science and Biotechnologies, University of Agricultural Sciences and Veterinary Medicine, Cluj-Napoca, Romania; 2 Faculty of Food Engineering, Tourism and Environmental Protection, Research Center in Technical and Natural Sciences, "Aurel Vlaicu" University, Arad, Romania; 3 INCDO-INOE 2000, Subsidiary Research Institute for Analytical Instrumentation, Cluj-Napoca, Romania; 4 Veterinary Sciences Division, Bacteriology Branch, Agri-Food and Biosciences Institute, Belfast, United Kingdom; 5 Faculty of Infectious & Tropical Diseases, London School of Hygiene and Tropical Medicine, London, United Kingdom; Guru Angad Dev Veterinary and Animal Sciences University, INDIA

## Abstract

Țaga cheese is a traditional Romanian smear-ripened cheese made from bovine milk and identified with the name of the village and caves where it is produced. As no previously reported microbiological and chemical studies have been undertaken on this product, this research aimed to investigate the microbiological and biochemical characteristics which ensure the uniqueness of Țaga cheese during the ripening process, to inform producers as to key quality determinants. Cheese samples, consisting of retail blocks, were collected on days 2, 5, 12, 18, and 25 of the ripening process. The evolution of lactic microbiota during the production and maturation of traditional cheeses involves isolating lactic acid microorganisms present in cheese. Cheese samples were analyzed for pH, fat, NaCl, fatty acids, and volatile compounds. The microbial ecosystem naturally changes during the maturation process, leading to variation in the microorganisms involved during ripening. Our results show that specific bacteria were identified in high levels during the entire ripening process and may be responsible for milk fat lipolysis contributing directly to cheese flavor by imparting detailed fatty acid flavor notes, or indirectly as precursors formation of other flavor compounds.

## Introduction

Natural cheese varieties contain microorganisms that are essential during manufacture and ripening,. These microorganisms can be divided into primary or secondary microbiota depending on the type of cheese. Still, ultimately they are responsible for the development of both flavor and texture [1]. The taste and texture of cheese result from a biochemical interaction between the microorganisms, the milk enzyme composition of milk, and the ripening conditions used by different processors [2].

Țaga cheese, also known as Năsal cheese, is a raw milk cheese named after one of the villages in the Transilvania region of Romania. It is an acid-curd cheese with a creamy texture, exhibiting a very strong pungent flavor with a slightly spicy taste [3]. Currently, it is produced from cow's milk by the Someș Arieș Agricultural Cooperative and it is certified as a traditional Romanian product. Approximately 51100 kg of Țaga cheese is produced annually. Locally, this cheese is considered unique because its maturation takes place in a special microbiological environment from the natural cave from Țaga. The caves' conditions are typical temperatures of 14–15°C and an air relative humidity of 90–95%, mostly constant throughout the year. These conditions could potentially favor the development of a specific selection of bacteria, such as *Brevibacterium linens* during the ripening process which may be responsible for the transformation of the cheese into a highly appreciated specialty by the costumers.

Mihaiu et al. [3] studied the indigenous microbial composition of the cave walls and reported that the bacteria identified consisted of 45% *Micrococcus* spp., 45% *Corynebacterium* spp. and 10% *Brevibacterium linens*. However, no detailed chemical or microbiological analysis of Țaga cheese has been reported. Raw milk cheeses can benefit organoleptically from the rich and diverse microbiota in the milk from which they are made [4]. Still, consumer safety must also be considered due to pathogenic bacteria's potential risks [4, 5]. In most cheeses, the use of pasteurized milk and starter cultures of lactic acid bacteria (LAB) ensures product safety [6]. However, many traditional cheeses rely on the endogenous microbiota of the milk [7].

Regardless of the LAB source, a series of complex physical, chemical and microbiological changes occur during the ripening process. The specific microbiota plays an essential role in forming flavor compounds and texture modification [8, 9]. The biochemical changes occurring during ripening include the metabolism of residual lactose and lactate,citrate, lipolysis and proteolysis and are followed by secondary biochemical changes, which are very important for the development of many volatile flavor compounds and include the metabolism of fatty acids and amino acids [10–12].

Țaga cheese is manufactured in an artisanal manner using raw bovine milk produced in the Taga area (Transylvania, Romania) and supplied by local farmers from Someș Arieș Agricultural Co-operative. The production process consists of collecting and shipping raw bovine milk to a dairy geographically close to the caves where the cheese will be matured. Before using, the milk is filtered, in order to eliminate the posible physical impurities, then analyzed to determine its quality (density, acidity, protein, and fat content). A single batch of cheese is produced each day, with the milk being standardized to 2.7–3.1% fat content before use. The milk is coagulated by heating it to 29–30°C, adding rennet by stirring the milk for 1–2 minutes with the help of stirrers, after which the movement of the milk is stopped, then waiting for 30–35 minutes. Curd processing is initially made by cutting and chopping, The curd is cut into cubes with a side of 5–6 cm, for 3–5 minutes; The cutting is done with the help of harps mounted on the arms of the mechanized valve shakers. The curd is then continuously blended for 15–20 minutes and mixed with salt, in a proportion of 1% (w/w) relative to the curd weight. The curd is then heated to 45°C and mixed for 5–8 minutes to obtain an overall temperature distribution of 34–45°C within the whole mass. Finally, the curd is left to rest for approximately 10 minutes to dehydrate and increase the whey acidity to 11–12° Thörner (T).

After proper dehydration of the curd in the mechanized valve, the curd is introduced in parallelepiped shapes with a height of 4 cm, length 22 cm and width of 12 cm, lightly pressed by hand then inserted into the mechanized press. The cheese is made in two types of weight: 0.6 kg and 0.3 kg. When 0.3 kg of cheese is made, before it is put into the mold, each half is cut in half. A sufficient number of turns are applied to the 4 quarters so that each piece forms a shell on all sides. During this procedure, the temperature is kept between 18–20°C.

The resulting molded 'bricks' undergo salting by storing them in brine with a concentration of 16–18% (w/v) at 14–15°C for 4–6 hours. After salting, the cheese is left to dry on shelves for 4–16 h, then transferred to the cave for maturation. The temperature is approximately 14–15°C in the cave, and air humidity of 90–95% is relatively constant throughout the year.

Maturation takes 25–30 days, and during this period, the cheeses are turned over daily during the first week, and subsequently every 2–3 days. The cheeses are washed with brine at these times, with a concentration of 10% (w/v). During this time, the surface coloration develops with characteristic surface bacteria. Once the maturation process is completed, the cheeses are again washed, then left to dry. Then the cheese surface is dusted with starch powder, and it is packed in parchment paper. The amount of Țaga cheese that is produced daily is approximatively 140 kg.

Despite the heterogeneity of the milk composition and microbiota and the ripening process's complexity, the producers of Țaga cheese report a relatively consistent product. To more fully understand the processes by which the mature product is obtained, this study was undertaken. Its main objective was to enumerate critical groups of microorganisms during the maturation process, usually about 25 days, to contribute to the maturation process and product safety. Further, the levels of fatty acids were measured, and key parameters were pH, salt content, fat content, and volatile compounds. Their contribution to the final product could be assessed by evaluating the changes undergone by these parameters during the maturation process.

As neither chemical nor microbial extended studies on Țaga cheese have been published, this study's main objective was to undertake microbiological and biochemical analyses of parameters that may ensure the uniqueness of Țaga cheese and evaluate their changes during the ripening process. The microbial studies on the cheese would also serve to assess its food safety.

## Materials and methods

### Sampling

For this study, Țaga cheese samples were collected directly from the manufacturer, Someș Arieș Agricultural Co-operative, andsampling was undertaken 2, 5, 12, 18, and 25 days after cheese production initiation. Each sample set consisted of three 'bricks' of cheese (each approximately 300 g) taken from one day's cheese production, i.e., from a single batch of milk and the same vat. The three samples were collected in individual sterile plastic bags, deposited in the laboratory's refrigerator at 4°C and collected on the same day for shipment to the analytical laboratory under chilled conditions. Analyses were initiated on the day of sampling. Each set of three samples were subjected to all analyses; hence they were all undertaken in triplicate.

### Microbiological analyses

Two samples were aseptically removed from each cheese 'brick' for analysis. One sample was representative of the cheese surface and the other of the core; the latter sample is subsequently referred to as the 'deep' sample. Each sample consisted of 25 g of material which was homogenized with 225 mL of a sterile solution of 2% sodium citrate (Nordic Chemicals, Cluj, Romania) at 40–45°C, for 2 minutes, using a stomacher (BagMixer®400, Interscience, Paris, France), thus making a 1/10 dilution. Further decimal dilutions were prepared by mixing 10 mL of the previous dilution with 90 mL of buffered peptone water (Merck, Darmstadt, Germany).

Bacteria were enumerated using the following media and incubation conditions: Plate Count Skim Milk agar media (Merck, Darmstadt, Germany) was used for determining mesophilic bacteria (48 h, 30°C) and also psychrotrophic bacteria (7 days, 7°C); DeMan, Rogosa, and Sharpe agar, (Liofilchem, Roseto degli Abruzzi, Italy) (5 days, 30°C) for *Lactobacillus* spp. and the same medium was used for *Leuconostoc* spp. (4 days, 22–25°C); M17 agar (Liofilchem, Roseto degli Abruzzi, Italy) was used for *Lactococcus* spp. (18-24h, 30°C); selective agar No. 110 acc. to Chapman (Merck, Darmstadt, Germany) was used for *Micrococcus* spp.(18-24h, 30°C); Kanamycin Aesculin Azide agar (KAAA, Liofilchem, Roseto degli Abruzzi, Italy) was used for enterococci (18–24 h, 37°C) and Violet Red Bile Glucose Agar (VRBGA, Liofilchem, Roseto degli Abruzzi, Italy) (18–24 h, 37°C) was used for enterobacteria. Besides, fungi were enumerated on Oxytetracycline Glucose Yeast Extract agar (Liofilchem, Roseto degli Abruzzi, Italy) (5 days, 22°C). It should be noted that no further identification of the colonies obtained on the selective media was undertaken; hence all counts obtained are presumptive identifications based on the particular properties of the media (ICMSF).

Two different inoculation techniques were applied [3]. Pour plates were prepared using 1 mL of each dilution to determine the total number of mesophilic bacteria, psychrotrophic bacteria, lactobacilli, enterococci, enterobacteria, and fungi. Surface plating, using 0.1 mL of each dilution, was used to determine the number of lactococci, micrococci, and *Leuconostoc* bacteria. Triplicate platings on each medium were made, and the average value was represented as log10 CFU/g.

### Physico-chemical analyses

Samples were analyzed in triplicate for pH measurement, 10 g of sample was homogenized in 10 mL of distilled water, and the pH of the resultant slurry was measured using a digital pH meter (basic pH Meter PB10, Sartorius, Göttingen, Germania). The dry matter was determinated according to AOAC 926.08. The fat content of the cheese was determined according to the Van Gulik method. Briefly, the determination consists of dissolving the protein with sulphuric acid, followed by separation of the cheese fat in a Van Gulik butyrometer by centrifugation, the separation being assisted by adding a small quality of amyl alcohol. The fat content is read directly on the butyrometer scale. NaCl was determined using the potentiometric titration method described in IDF standard 17A (International Dairy Federation).

### Analysis of fatty acid contents

To determine the fatty acid content, 10 grams of cheese/10 mL milk was extracted with 25 mL diethyl ether at room temperature for 2 hours. The mixture has been centrifuged for 5 minutes at 6700 g. The fatty upper-layer has been separated, and the fatty acids have been isolated using the method described in Copolovici et al. 2017 [13]. Briefly, the fatty acids have been transmethylated into corresponded fatty acid methyl esters using methanol/toluene/sulphuric acid mixture (88/10/2 v/v/v). The resulting methyl esters extracted twice with n-heptane and analyzed by GC-MS (Shimadzu 2010 Plus). The constituents have been identified based on fatty acid standards and NIST 14 and Wiley 09 mass spectra libraries.

### Qualitative analysis of volatile components

HS-SPME analysis was performed using an Agilent 6890A (Agilent, USA) Gas Chromatograph coupled to a Mass Spectrometer 5975 (Agilent, USA) and equipped with an HP-5MS column (30 m; 0.25 mm i.d.; 0.25 μm film thickness, Restek, Bellefonte, PA, USA). The oven temperature program was: from 40°C, hold 5 min, to 100°C at 5°C/min, then from 100°C to 200°C at 10°C/min and finally hold for 10 min. The GC injector was set at 250°C, and the injections were performed in splitless mode. The carrier gas was helium at the constant flow of 1 mL^−1^. The transfer line to the mass spectrometer was maintained at 230°C, and the ion source temperature was set at 250°C. The mass spectra were obtained using electron impact mode (70 eV) by collecting the data at a rate of 1 scan s−1 over the m/z range of 45–450.

### Statistical analysis

All measurements were performed in triplicate, and the results were represented as mean ± SEM. Statistical analyses were performed with the GraphPad Prism 8 statistics program. Data statistical analyses were achieved by using one-way ANOVA and Tukey-test. The level of significance was set at p < 0.05.

## Results

### Microbial changes during the ripening process of Țaga cheese

Within the microbial population studied (Table 1), three patterns of change can be seen during the maturation process, and these occur both in the surface samples and in the core samples.

**Table 1:** Effect of the ripening process of Țaga cheese on the microbial population studied.

	Day of ripening										
Microorganism group		2		5		12		18		25	
	Medium	S	D	S	D	S	D	S	D	S	D
Total Mesophilic	**PCA** 30°C / 48 h	10.85±0.22^a^	8.40±0.54^a^	8.20±0.47^b^	7.08±0.31^b^	8.51±0.63^c^	7.08±0.22^b^	8.46±1.07^d^	7.41±0.68^c^	8.38±0.12^e^	7.08±1.25^b^
Total Psychrotrophic	**PCA** 7°C / 10 days	8.65±0.93^a^	8.58±0.81^a^	8.46±0.16^b^	7.18±0.64^b^	7.10±0.49^c^	6.81±1.05^c^	7.33±0.22^d^	6.58±0.0.23^d^	7.13±0.22^c^	6.74±1.75^c^
Lactococci	**M 17** 30°C / 24h	7.30±0.77^a^	8.38±1.22^a^	6.23±1.00^b^	6.40±0.59^b^	5.76±1.03^c^	6.22±0.35^c^	4.99±0.87^d^	5.13±0.51^d^	3.81±0.26^e^	4.10±0.49^e^
Leuconostoc	**MRS**22-25°C / 4 days	6.11±0.51^a^	5.99±0.61^a^	5.41±0.14^b^	5.43±0.89^b^	5.10±0.11^b^	5.42±0.31^b^	5.17±0.10^b^	5.22±0.54^b^^,^^c^	5.05±0.22^b^	4.98±0.41^c^
Lactobacilli	**MRS** 30°C / 5 days	7.82±0.45^c^	7.95±0.62^b^	7.88±0.51^c^	7.98±0.22^b^	8.40±0.85^a^	8.47±0.38^a^	8.30±0.69^a^	8.31±0.80^a^	8.16±0.84^b^	8.40±0.34^a^
Micrococci	**CHAPMAN** 30°C / 24h	4.08±0.51^a^	4.40±0.37^a^	3.54±0.54^b^	3.08±0.52^b^	3.39±0.28^c^	3.38±0.52^c^	1.54±0.34^d^	nd	1.36±0.28^e^	nd
Enterococci	**KAA** 37°C / 24h	5.43±0.23^a^	5.27±0.39^a^	4.39±0.29^b^	3.93±0.63^b^	2.51±0.55^c^	1.75±0.19^c^	1.58±0.23^d^	nd	1.72±0.15^e^	nd
Enterobacteria	**VRBGA** 37°C / 24h	8.85±0.12^a^	8.40±0.36^a^	6.20±0.60^b^	6.08±1.01^b^	4.14±0.14^c^	1.79±0.47^c^	1.79±0.16^d^	nd	nd	nd
Fungi	**OGYE** 22°C / 5 days	4.54±0.25^b^	4.45±0.30^b^	4.77±0.15^c^	4.62±0.21^b^^,^^c^	4.95±0.5^d^	4.79±0.13^c^	5.10±0.49^d^	5.01±0.53^a^	5.39±0.35^a^	5.27±0.41^a^

^a-e^, Different superscript letters within the same row indicate significant differences (p ≤ 0.05)

PCA, plate count, skim milk agar media; M17 agar; MRS, DeMan, Rogosa, and Sharpe agar; Chapman, selective agar No. 110 acc. to Chapman; KAA, kanamycin aesculin azide agar; VRBGA, violet red bile glucose agar; OGYE, oxytetracycline glucose yeast extract agar. S- surface sample, D- deep sample, nd- not detected

The mesophilic bacteria population showed a gradual but significant (p ≤ 0.05) decrease from 10.85 log CFU/g at the beginning of the ripening process to 8.38 log CFU/g at the end of the ripening process. The psychrotrophic microbial population also showed a significant (p ≤ 0.05), but slightly less marked decrease, falling from 8.65 logs CFU/g to 7.13 log CFU/g at the end of the ripening process.

For the three groups of lactic acid bacteria, two distinct patterns of change were seen. The number of lactococci and leuconostoc fell significantly (p ≤ 0.05) during the ripening process, from 7.30 6.11 log CFU/g, respectively, to 3.81 and 5.05 log CFU/g. In marked contrast to this, lactobacilli became the dominant LAB during ripening, with numbers of 7.82 log CFU/g after 2 days, rising to 8.16 log CFU/g at the end of the ripening period.

The microbiota represented by micrococci, enterococci, and enterobacteria showed a very significant reduction (p ≤ 0.05) during ripening, falling to 1.36 log CFU/g for micrococci and 1.72 log CFU/g for enterococci in the surface samples. However, they were below the limit of detection in the core samples, i.e., less than 1.0 log CFU/g. In the case of enterobacteria, at the end of maturation, they were below the limit of detection (1.0 log CFU/g) in both surface and core samples

Considering the yeasts and molds (YAM), a slight, but significant (p ≤ 0.05) increase in numbers was seen in both core and surface samples during maturation. Their presence is being maintained by the appropriate ripening conditions, such as high humidity and constant temperature.

### Changes in physicochemical parameters during ripening

The changes in the physicochemical parameters obtained throughout the ripening of Țaga cheese are shown in Table 2.

**Table 2:** Changes in salt content, fat content, and pH during the ripening process.

Day of ripening					
	2	5	15	18	25
**NaCl (%)**	2.25±0.25^a^	2.55±0.38^a^	2.65±0.10^a^	2.00±0.22^a^	2.60±0.20^a^
**FAT (%)**	22.50±0.76^a^^,^^c^	22.50±0.62^a^	21.75±0.54^b^^,^^c^	22.84±0.81^a^	22.02±0.61^a^^,^^b^
**pH**	5.45±0.20^a^	5.40±0.20^a^	5.50±0.42^a^	5.58±0.23^a^	5.65±0.16^a^
**Dry matter (%)**	42.25±1.25	42.85±1.19	44.62±1.35	45.63±1.41	46.13±1.68

a-c, Different superscript letters within the same row indicate significant differences (p ≤ 0.05); Fat and salt are expressed as percentages.

Comparing to milk, the pH underwent a rapid fall during cheeses manufacture, reaching values of about 5.45 in the 2-day old cheese and remains in a range of 5.5–5.6 on the ripening process. Salt quantity in Țaga cheese varies between 2.25–2.65%. In comparison, the amount of fats depends on the milk's quality and remains around the value of 22, with limits between 22.02 and 22.84% during the entire process of ripening.

### Change of fatty acids contents

We have next investigated the fatty acid composition as described in material and methods section. The fatty acids (FA) identified at different stages of storage of the cheese are shown in Table 3.

**Table 3:** Fatty acids detected during the ripening process (nmol/g).

Fatty acids		Cheese day of ripening				
		2	5	12	18	25
C10:0	Capric acid	13.86^a^	14.20^a^	6.87^b^	7.07^c^	6.80^b^
C12:0	Lauric acid	18.52^a^	18.86^a^	10.16^b^	10.74^c^	9.83^d^
C14:1	Myristoleic acid	0.45^e^	0.50^a^	0.24^b^	0.23^c^	0.21^d^
C14:0	Myristic acid	52.56^a^	51.85^b^	27.22^c^	28.50^d^	26.52^e^
C15:1	Pentadecenoic acid	2.50^a^	2.68^a^	1.16^b^	1.47^c^	1.31^d^
C15:0	Pentadecylic acid	5.52^d^	6.30^a^	2.11^b^	2.62^c^	2.56^c^
C16:1	Palmitoleic acid	7.32^e^	7.62^a^	3.33^b^	3.48^c^	3.23^d^
C16:0	Palmitic acid	148.25^a^	144.00^b^	73.06^c^	78.62^d^	74.34^e^
C17:1	Heptadecenoic acid	5.05^a^	5.55^b^	2.62^c^	2.69^d^	2.53^e^
C17:0	Margaric acid	3.05^a^	3.10^a^	1.40^b^	1.59^c^	1.37^b^
C18:2	Linoleic acid	2.13^a^	1.67^b^	1.41^c^	1.06^d^	1.21^e^
C18:1	Oleic acid	124.86^e^	135.15^a^	57.72^b^	61.58^c^	55.43^d^
C18:0	Stearic acid	59.56^e^	66.05^a^	26.33^b^	29.41^c^	26.64^d^
C19:1	Nonadecenoic acid	0.32^c^	0.41^a^	0.16^b^	0.15^b^	0.13^b^
C19:0	Nonadecylic acid	0.33^a^	0.29^b^	0.19^c^	0.17^d^	0.24^e^
C20:1ω3	(17Z)-17-icosenoic acid	0.75^e^	0.81^a^	0.31^b^	0.38^c^	0.55^d^
C20:1ω6	(14Z)-14-icosenoic acid	0.42^e^	0.57^a^	0.21^b^	0.10^c^	0.17^d^
C20:1ω9	Gondoic acid	1.49^d^	1.60^a^	1.45^b^	1.41^c^	1.43^b^^,^^c^^,^^d^
C20:0	Arachidic acid	0.63^c^	0.71^a^	0.20^b^	0.23^b^	0.20^b^
C22:1ω6	(16Z)-16-docosenoic acid	1.55^d^	1.61^e^	3.13^a^	2.65^b^	2.84^c^
C22:1ω9	Erucic acid	0.27^d^	0.29^d^	0.11^a^	0.09^b^	0.08^c^
C22:0	Behenic acid	0.31^a^	0.31^a^	0.15^b^	0.12^b^	0.15^b^
C23:0	Tricosylic acid	13.86^e^	14.20^a^	6.87^b^	7.07^c^	6.80^d^
MCFA		32.38	33.06	17.03	17.81	16.63
LCFA		415.17	428.86	199.12	213.69	198.07
VLCFA		2.71	2.64	3.58	3.13	3.31
**Total FA**		**452.26**	**469.56**	**231.73**	**252.64**	**243.01**
MUFA		144.98	156.79	70.44	74.23	67.91
PUFA		2.13	1.67	1.41	1.06	1.21
SFA		303.16	306.08	147.87	159.35	148.90
UFA		147.09	158.47	71.86	75.29	69.12
SFA/UFA		2.15	2.02	2.15	2.21	2.25
PUFA/MUFA		0.01	0.01	0.02	0.01	0.02

nd- not detected

MCFA, medium-chain fatty acids; LCFA, long-chain fatty acids; VLCFA, very-long-chain fatty acids, MUFA = Monounsaturated fatty acids, PUFA = Polyunsaturated fatty acids, SFA = Saturated fatty acids, UFA = Unsaturated fatty acids.

^a-d^, Different superscript letters within the same row indicate significant differences (p ≤ 0.05)

The milk analysis was performed to observe the correlation with the cheese composition and revealed that the most abundant fatty acids are C16:0 (palmitic acid) 21.99±0.37 nmol/mL, followed by oleic acid (C18:1) 17.88±0.09 nmol/mL. Significant quantities were observed for stearic acid (C18:0) 8.43±0.28 nmol/mL and myristic acid (C14:0) 6.26±0.11 nmol/mL. These fatty acids are followed by lauric (C12:0) and capric acid (C10:0) with 3.62±0.19 nmol/mL and 2.37± 0.09 nmol/mL, respectively.

The other fatty acids observed in milk are below 1 nmol/mL, include palmitoleic acid (C16:1) 0.97±0.01 nmol/mL, linoleic acid (C18:2) 0.67±0.02 nmol/mL, heptadecenoic acid 0.58±0.03 nmol/mL, pentadecylic acid (C15:0), 0.48±0.01 nmol/mL, margaric acid (C17:0) 0.29±0.03 nmol/mL, pentadecenoic acid (C15:1) 0.26±0.01 nmol/mL and myristoleic acid (C14:1) 0.24±0.01 nmol/mL.

The analysis of cheese, Table 3, revealed that the majority of fatty acids were saturated, with C16:0 (palmitic acid) being the most abundant. Other acids following this fatty acid are oleic acid (C18:1), stearic acid (C18:0), and myristic acid (C14:0). This may be due to large amounts of palmitic, oleic, stearic, and myristic acid in milk fat. Milk fat generally contains approximately 67.82% saturated fatty acids (SFA), similar with the SFA content from cheese samples during maturation (68.29% at the end of maturation). The ratio between SFA and UFA is increasing from around 2 in the first days of ripening to 2.25 on the last day.

**Fig 1:**
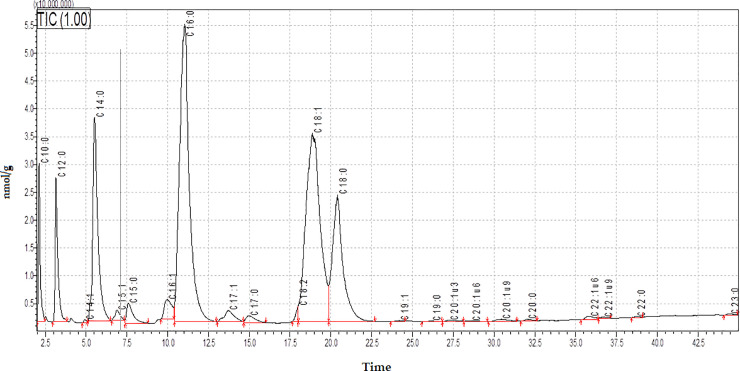
GC-MS profile of fatty acids from cheese (day 5). C10:0-Capric acid, C12:0-Lauric acid,C14:1-Myristoleic acid, C14:0-Myristic acid, C15:1-Pentadecenoic acid, C15:0-Pentadecylic acid, C16:1-Palmitoleic acid, C16:0-Palmitic acid, C17:1-Heptadecenoic acid, C17:0-Margaric acid, C18:2-Linoleic acid, C18:1-Oleic acid, C18:0-Stearic acid, C19:1-Nonadecenoic acid, C19:0-Nonadecylic acid, C20:1ω3-(17Z)-17-icosenoic acid, C20:1ω6-(14Z)-14-icosenoic acid, C20:1ω9-Gondoic acid, C20:0-Arachidic acid, C22:1ω6-(16Z)-16-docosenoic acid, C22:1ω9-Erucic acid, C22:0-Behenic acid, C23:0-Tricosylic acid.

The medium-chain fatty acids detected were capric and lauric acids (C10:0, C12:0) and represent 33,61 nmol/g in milk, 32,38 nmol/g in unmatured cheese (day 2), and 16,63 nmol/g în the final product. Long-chain fatty acids represent the majority of the fatty acids content with oleic (C18:1), stearic acid (C18:0), and myristic acid (C14:0) being the most important quantitatively. Still, more other fatty acids are present in significant quantity palmitoleic acid (C16:1), pentadecylic acid (C15:0), pentadecenoic acid (C15:1), heptadecenoic acid (C17:1), linoleic acid (C18:2). We found that medium and long chain fatty acids are predominant in our cheese samples and have the same pattern in our cheese samples. In contrast, very long fatty acids show a different pattern during ripening, but these FA concentrations were very low (Fig 1).

At the end of the ripening, the quantity of long and medium-chain fatty acids in Țaga cheese was significant, with palmitic acid (C16:0) being the most abundant (74.34 nmol/g), followed by oleic acid (C18:1) (55.43 nmol/g), stearic acid (C18:0) (26.64 nmol/g) and myristic acid (C14:0) (26.52 nmol/g).

### Change of volatile components

The volatile organic compounds (VOCs) of the cheese samples and the results are depicted in Table 4.

**Table 4:** Volatile components evolution during the ripening process (%).

Class of compounds	Compounds	Day of ripening				
		2	5	12	18	25
Alcohol	1-hexanol	1.9±0.10^a^	2.1±0.15^a^	2.3±0.18^a^	1.9±0.10^a^	2.2±0.17^a^
aldehyde	2-methyl butanal	0.09±0.12^b^	0.14±0.10^c^	0.18±0.26^a^^,^^c^	0.21±0.11^a^	0.22±0.10^a^
Ketone	2-butanone	0.27±0.18^b^	0.51±0.09^a^	0.32±0.10^b^	0.44±0.12^a^^,^^b^	0.35±0.08^b^
Ketone	2-hexanone	1.9±0.20^b^	3.5±0.26^c^	2.1±0.18^b^	1.9±0.12^b^	4.8±0.34^a^
Ester	ethyl isovalerate	4.5±0.41^c^	0.95±0.22^d^	7.2±0.20^a^	1.8±0.10^b^	1.3±0.10^b^^,^^d^
aldehyde	octanal	11.8±0.50^b^^,^^d^	12.1±0.35^d^	20.3±0.62^a^	11.4±0.37^b^	15.1±0.40^c^
aldehyde	2-octenal	2.1±0.15^b^	2.44±0.20^b^	2.2±0.22^b^	3.5±0.48^a^	3.7±0.12^a^

^a-d^, Different superscript letters within the same row indicate significant differences (p ≤ 0.05)

Aldehyde octanal was the compound found in the highest amount in the analyzed samples. High levels of alcohol 1-hexanol and ketone 2-hexanone were also present in the cheese samples. The Other volatile organic compounds identified were the aldehydes 2-methyl butanal and 2-octenal, the ketone 2-butanone, and the ester ethyl isovalerate. The most abundant volatile components were the aldehyde octanal and the ester ethyl isovalerate, followed by aldehyde 2- octenal. Values were highest for the octenal in day 15 (20.3±0.62), but the high value of this aldehyde is also observed in day 18 and 25 of ripening, 11.4% 15.1%, respectively. A constant increase is kept in the case of aldehyde 2-methyl butanal starting from 0.09±0.12% in day 2 and reaching 0.22±0.10% in the last stage of the ripening process.

## Discussion

Țaga cheese is a creamy texture acid-curd cheese, manufactured with raw bovine milk having a very strong flavor and a light spicy taste. Regular brine washing of the surface resulted in the surface showing coloration indicative of a pigmented microbial flora and appears to have an increased number of mesophiles on the surface, relative to the core, Table 1. However, for most of the surface and core samples taken, there was no significant difference between them. Marshall [14] emphasized the importance of rapid fermentation to product quality as the production of lactic acid, bacteriocins, and other antagonists all act to prevent the growth of spoilage and pathogenic bacteria [15], and *Lactobacillus* spp. usually fulfill this role [7].

Even if the pH of Țaga cheese remain almost stable during the ripening process a decrease of total mesophilic and psychrotrophic bacteria was registered, this may be attributed to the increase in dry matter of the final product, oxygen deficiency or various secondary metabolites produced by the microorganisms.

In this study, enterobacteria were used as indicator bacteria for the presence of pathogens such as *Salmonella* and pathogenic *Escherichia coli*, and both surface and core samples showed an absence of enterobacteria in the final product. These pathogens can potentially be present in raw bovine milk [4]. Again, numbers were reduced to undetectable in core samples, albeit with low numbers detected in the surface samples. Given the latter's prevalence on human skin [16] and the handling the cheese undergoes during regular brininga number of micrococci may arise from cross-contamination. Enterococci have also been reported in high numbers in raw milk cheeses by other authors [17, 18]. The rapid disappearance of *Enterobacteriaceae* during ripening may be associated with the decrease of moisture and of pH of the cheese compared with compared to milk, lack of oxygen, depletion of sugars by lactic acid bacteria and the secondary microflora activity. Overall, the cheese microbiota can be seen to eliminate potential pathogens during the maturation process, as has been noted with other such products [7], ensuring a safe product.

Considering the surface coloration which develops, *Brevibacterium linens*, previously identified as part of the endogenous flora of the Taga cave, is known to cause a similar coloration on the surface of a variety of smear surface-ripened cheeses, such as Limburger, Münster, Brick, Tilsiter, and Appenzeller [19]. Hence, it may merit detailed study in the future, as the cheeses' surface is considered a significant characteristic [20]. The red-orange color of the cheese's smear is primarily due to the pigments produced by *Brevibacterium* spp., *Corynebacterium* spp., *Micrococcus* spp., and *Arthrobacter* spp. [21], three of these genera (*Brevibacterium* spp., *Corynebacterium* spp., *Micrococcus* spp.) are reported in the Taga cave [3].

During the ripening process, a series of complex physical, chemical, and microbiological variations occur within cheese [1]. The biochemical changes occurring during the ripening process include the metabolism of residual lactose and lactate and citrate, lipolysis and proteolysis and are followed by secondary biochemical changes, which are very important for the development of many volatile flavor compounds and include the metabolism of fatty acids and amino acids [12, 22]. Although numbers of lactococci, enterococci, and micrococci fall throughout maturation, Table 1, the dead organisms can release enzymes that can catalyze, for example, peptidolytic processes, which are essential for flavor development [1].

Lipolysis is a key biochemical process during the cheese manufacturing process, directly impacting the flavor development and microbiology during the ripening process [23]. Five of the bacteria studied; lactococci, leuconostoc, lactobacilli, enterococci, and micrococci can participate in the cheeses' ripening development proteolytic, lipolytic, and esterolytic activities[24–26]. Also, as noted above, even dead micrococci can contribute to ripening as their extracellular lipase can remain active [27]. However, the lactobacilli, Table 1, are generally accepted as playing a significant role during maturation [1]. This microbial group will increase the concentration of small peptides, free amino acids, and free fatty acids [28]. The slower metabolism of lactobacilli and their capacity to adapt to adverse conditions (acidity, higher concentrations of NaCl) could contribute to their predominance in the last stage of ripening [15].

Being resistant to the salt content of cheese, lactobacilli contributes to the safety and microbiological quality due to a reduction in pH [29, 30]. The salt content of Țaga cheese varies between 2.25–2.65% and leads to a consequently appropriate microbiota, possibly including *Brevibacterium linens*, characterized by a relatively high salt tolerance (15%) [19]. The cheese's initial fat concentration depends on the quality of the milk. In this study, it remained relatively constant around the value of 22% with limits between 22.02% and 22.84% during the entire ripening process. Thus there was no apparent degradation of this component of the cheese during maturation.

Cow's milk is the richest source of oleic acid (24%), while its content in goat and sheep milk is, on average, 18% of all fatty acids [31]. The milk used for making Taga cheese is close to these data; the oleic acid (C18:1) represent 21,99% of total fatty acids. However, some authors reported higher concentration (more than 20% of all fatty acids) in sheep and goat milk [32]. In ruminant's milk, there are also relatively small but significant contributions from other monounsaturated fatty acids (MUFA) such as myristoleic acid (C14:1) (about 1%), palmitoleic acid (C16:1) (about 1.5%), and very desirable vaccenic acid, which is a precursor of conjugated linoleic acid (CLA) in the human organism (1.5%-5%) [31]. The profile of milk fatty acids influences the profile of fatty acids in cheese, so we decided to analyze both the samples during the non-maturing process and the milk used as raw material. In our milk sample the myristoleic acid (0.24 nmol/g), palmitoleic acid (0,97 nmol/g) were present along with pentadecenoic acid (C15:1) (0,26 nmol/g) and heptadecenoic acid (C17:1) (0,58 nmol/g).

Ruminant diets have a direct influence on the fatty acid (FA) profile of raw milk when the animals have direct access to pasture, increasing the presence of the beneficial polyunsaturated fatty acids (PUFA) [33]. The cheese obtained from the milk of these cows is preferred by consumers [34]. Different varieties of cheese have showed different levels of lipolysis from moderate (~ 0.3–0.5 g FA / 100 g fat) to extensive (up to 20 g FA / 100 g fat). The level of lipolysis is influenced by microbial agents with lipolytic potential that are present in the cheese [35]. Extensive lipolysis is considered undesirable, mostly short chain FA production, which may directly affect flavor development [36]. Furthermore, the pH prevailing in cheese exerts a considerable influence on FA's flavor impact; by day 18 of ripening, pH is ∼5.6 (Table 2). Hence, a substantial portion of FA is present in salt form and has little impact on aroma [37].

Țaga cheese is a traditional cheese made in small quantities. After the literature analysis, no research was found related to determining the fatty acid profile during its production and storage stages. For this reason, the results obtained from this research will be interpreted, taking into consideration the studies conducted in other similar cheese varieties. This study shows the profile of fatty acids during Țaga cheese ripening (Table 3).

Most studies reveal the accumulation of fatty acids in cheese, during long periods of maturation. However, there are other studies, about traditional cheeses, with short maturation periods, that show a decrease in fatty acid content, although initially the profile of fatty acids it was similar to that of milk.

The level of the fatty acids gradually increases according to the maturation; this growth being observed after 60 or even 90 days of maturation [38, 39]. Asya et al. [38] studied traditional Turkish cheese, named Kars Gravyer Cheese, which presents the same pattern as Taga cheese, but the maturation period is more extended (90 days). Double carbon saturated and unsaturated 16 fatty acids were identified in Kars Gravyer Cheese samples. According to the analysis results, the highest fatty acids in the cheese samples, like in Taga cheese, were palmitic, oleic, stearic, and myristic fatty acids [38]. In Kars Gravyer Cheese samples, the number of volatile fatty acids (C4: 0-C14: 0) increased to 25th day and decreased from 45th day to 90th day. While levels of free fatty acids (C16: 0-C18: 2) generally decrease at day 25, they have begun to increase again until the end of the ripening period [38]. As for the other studies, some of the differences after the 30th day can be observed in the distribution of fatty acids during aging. These differences can result from different applications in cheese production technology. In the study of Kara et al. [39], Afyon Tulum Cheese has been reported to maintain a high percentage of unsaturated fat while maintaining low percentages of saturated fat, excluding C14:0—C18:0. The total average of unsaturated fatty acids has been reported to be 29.36 g / 100 g. Even though the ratio between SFA and UFA is increasing in the maturated cheese compared with a fresh one, the differences do not influence this cheese's dietary properties.

Many compounds with the same functional group originate from the same biochemical pathway of production in cheese [40]. In our study, the volatile organic compounds (VOC) found in the Țaga cheese, Table 4, were organic acids, alcohols, ketones, aldehydes, and esters. The latter arise from esterification of FA with ethanol, which can arise from lactose fermentation performed by several homofermentative bacteria, e.g., enterococci and lactobacilli, yeasts, and *Leuconostoc* spp. [41]. Aldehydes were shown to be produced from amino acids either by transamination followed by decarboxylation or by Strecker degradation [42] and can be easily reduced to alcohols [43]. Other volatile organic compounds identified here were the aldehydes 2-methyl butanal and 2-octenal, the ketone 2-butanone, and the ester ethyl isovalerate. Alcohols and ketones arise from oxidation and decarboxylation of fatty acids released during lipolysis.

The ratios of these FA and VOC components varies during ripening. The presence of the esters is probably related to esterase activity by lactic acid bacteria [44]. The 2-butanone can result from the reduction of acetoin by leuconostoc and lactobacillus [45], and both are present in Țaga cheese. Alcohols and ketones can arise from oxidation and decarboxylation of fatty acids released during lipolysis and are commonly produced by lactobacilli during milk fermentation [46]. The strong reducing conditions in cheese favored the formation of alcohols from aldehydes and ketones, following reaction pathways that involve alcohol dehydrogenases [47].

## Conclusions

Our study shows the microbiological and chemical composition of Țaga cheese. Because it is manufactured from raw bovine milk, the microbial load was relatively high at 2 days after the initiation of cheese production, especially in *Enterobacteriaceae*, which are known that may produce lipases breakdown milk fat, and could be potential pathogens, but their number decreased during the maturation process. However, the major bacteria groups found in cheese were: *Lactococi*, *Lactobacili* and *Leuconostoc*. The analysis of cheese, revealed that the majority of fatty acids were saturated, with C16:0 (palmitic acid) being the most abundant, but also MUFA and PUFA acids were identified in in small quantities. Aldehyde octanal was the compound found in the highest amount in the analyzed samples. High levels of alcohol 1-hexanol and ketone 2-hexanone were also present in the cheese samples. The ratios of fatty acids and VOC components varies during ripening. The presence of the esters is probably related to esterase activity by lactic acid bacteria. The 2-butanone can result from the reduction of acetoin by leuconostoc and lactobacillus, and both are present in Țaga cheese. The biochemical changes occurring during the ripening process include the metabolism of residual lactose, lactate, citrate, lipolysis, proteolysis and are followed by secondary biochemical changes, which are very important for the development of many volatile flavor compounds and include the metabolism of fatty acids and proteins.

Overall, the parameters derived over centuries to prepare this cheese have been proven effective. There is scope for further study to investigate the specific microbiota causing the cheese surface's characteristic coloration.
